# Mechanisms and therapeutic strategies of bidirectional crosstalk between hepatic stellate cell-derived cancer-associated fibroblasts and T cells in immune evasion and therapeutic resistance of hepatocellular carcinoma

**DOI:** 10.3389/fimmu.2026.1930718

**Published:** 2026-09-07

**Authors:** Kechuan Pan, Chunlian Ma

**Affiliations:** Department of Infectious Diseases, The First People's Hospital of Wenling, Affiliated Wenling Hospital, Wenzhou Medical University, Wenling, China

**Keywords:** cancer-associated fibroblasts, hepatic stellate cells, hepatocellular carcinoma, immune evasion, T cells, therapeutic resistance

## Abstract

Hepatocellular carcinoma (HCC) commonly develops in the context of chronic liver injury and fibrosis, and its immune evasion and therapeutic resistance are shaped not only by tumor-intrinsic factors but also by persistent interactions between the tumor stroma and immune cells. Hepatic stellate cells (HSCs) represent an important source of cancer-associated fibroblasts (CAFs) in HCC. HSC-derived CAFs can restrict the infiltration and function of effector T cells and promote the accumulation of immunosuppressive cells through CAF-associated and multicellular signaling networks, including TGF-β/SMAD, IL-6/STAT3, CXCL12/CXCR4, and CCL2/CCR2, as well as through extracellular matrix remodeling, vascular abnormalities, hypoxia, and metabolic reprogramming. Importantly, mediators such as IL-6 and CCL2 are not specific to HSC-derived CAFs and can also be produced by immune cells, hepatocytes, and other cellular populations within the HCC microenvironment. Conversely, cytokines released by distinct T-cell subsets, including IL-17A, TGF-β, IL-10, IFN-γ, and TNF-α, can in turn shape CAF activation, inflammatory states, and matrix-remodeling programs, thereby establishing a dynamic immune–stromal feedback loop. Focusing on the heterogeneity of CAFs and T cells, this review systematically summarizes the major mechanisms underlying bidirectional crosstalk between HSC-derived CAFs and T cells and their stage-specific roles in chronic liver injury, HCC initiation, progression, and therapeutic resistance. We further discuss potential therapeutic strategies involving CAF modulation, stromal remodeling, combination immunotherapy, and patient stratification. Current evidence suggests that this crosstalk axis may provide an important framework for understanding immune exclusion and therapeutic resistance in HCC. However, its clinical translation remains constrained by CAF heterogeneity, limited targeting specificity, insufficient causal evidence in humans, and safety concerns in the setting of underlying liver disease.

## 1 Introduction

Hepatocellular carcinoma (HCC) is one of the most common types of primary liver cancer and frequently arises in the setting of chronic liver disease and fibrosis (1–4). Persistent liver injury continuously drives inflammatory responses, hepatic stellate cell (HSC) activation, extracellular matrix (ECM) deposition, and remodeling of the immune microenvironment, thereby providing an important pathological basis for HCC initiation and progression. In recent years, immune checkpoint inhibitors have substantially reshaped the therapeutic landscape of advanced HCC; however, a considerable proportion of patients still exhibit primary or acquired resistance (5, 6). Because the efficacy of immunotherapy depends on effective infiltration and sustained function of effector T cells, the widespread restriction of T-cell infiltration, functional impairment, and exhaustion observed in HCC suggests that the tumor stroma and its interactions with T cells may represent important factors limiting responses to immunotherapy (7–10).

Cancer-associated fibroblasts (CAFs) are an important component of the stromal microenvironment in HCC (11). HSCs represent one of the major sources of CAFs in HCC, particularly in the context of chronic liver injury and fibrosis, where activated HSCs may progressively acquire CAF-like phenotypes. HSC-derived CAFs contribute to ECM remodeling and fibrotic stromal organization and may also influence T-cell infiltration, activation, and functional maintenance through multiple immunoregulatory mechanisms (12–14). At the same time, cytokines released by different T-cell subsets can in turn shape the activation state and immunoregulatory functions of CAFs (15, 16). However, because CAFs in HCC may arise from multiple stromal sources, not all CAF-related immunoregulatory findings can be attributed specifically to HSC-derived CAFs. The bidirectional crosstalk between HSC-derived CAFs and T cells should therefore be considered within the broader context of CAF heterogeneity and may represent an important mechanistic link connecting chronic liver injury, fibrotic stromal remodeling, HCC immune evasion, and therapeutic resistance.

Current research on HCC immunotherapy has largely focused on immune checkpoints, T-cell exhaustion, and myeloid cell-mediated immunosuppression, whereas existing studies and reviews have not yet systematically integrated the bidirectional interactions between HSC-derived CAFs and distinct T-cell subsets, their stage-specific evolution, and their relationship with therapeutic resistance (17, 18). Therefore, this review focuses on the bidirectional crosstalk between HSC-derived CAFs and T cells while explicitly distinguishing evidence derived from HSC-lineage CAFs from findings reported for CAFs more generally. We systematically summarize their cellular heterogeneity, molecular mechanisms, and dynamic roles across different stages of chronic liver injury, HCC initiation, progression, and therapeutic resistance. We further discuss their clinical relevance and potential therapeutic strategies, with the aim of providing a more integrated conceptual framework for understanding immune–stromal interactions and combination immunotherapy in HCC.

## 2 Remodeling characteristics of HSC-derived CAFs and T cells in HCC

Within the HCC tumor microenvironment, CAFs and T cells are key components of stromal remodeling and antitumor immunity, respectively. CAFs are not a uniform population in either origin or function but instead constitute a heterogeneous stromal ecosystem characterized by diverse cellular origins, activation states, and spatial distributions (19). HSCs are an important source of CAFs in HCC, particularly in the context of chronic liver injury and fibrosis, where persistent inflammation can drive HSC activation and the gradual acquisition of CAF-like features (20). However, CAFs may also arise from portal fibroblasts, mesenchymal stem cells, bone marrow-derived cells, and other potential stromal sources. HSC-derived CAFs should therefore be regarded as an important component of the HCC CAF ecosystem rather than as the entire CAF population or a population that is necessarily dominant.

CAFs also exhibit pronounced functional heterogeneity. Based on transcriptional and functional characteristics, they can be broadly categorized into myofibroblastic CAFs (myCAFs), characterized by matrix-producing molecules such as α-SMA and COL1A1; inflammatory CAFs (iCAFs), characterized by the secretion of inflammatory mediators such as IL-6 and CXCL8; and antigen-presenting CAFs (apCAFs), which express MHC class II-related molecules. More recently, single-cell sequencing and spatial transcriptomics have further identified CAF states with distinct spatial distributions and functional programs, including CAF-FAP, CAF-C7, HLA-DRB1^+^ CAFs, MMP11^+^ CAFs, and VEGFA^+^ CAFs (21). Some FAP^+^ CAF populations are associated with dense ECM and immune exclusion. CAF-FAP cells can form spatially organized stromal–immune ecosystems with PD-1^+^CD8^+^ T cells, whereas CAF-C7 cells are more closely associated with tissue-repair states at the tumor margin. VEGFA^+^ CAFs are predominantly enriched within the tumor interior and are associated with hypoxic and angiogenic programs (22–24). Different CAF states may therefore influence T cells through distinct mechanisms. Matrix-producing or FAP^+^ CAFs may primarily restrict CD8^+^ T-cell entry into the tumor parenchyma through ECM deposition and spatial barriers, whereas inflammation-associated CAFs may regulate T-cell recruitment and function through cytokine and chemokine networks. CAFs with antigen-presenting features may also participate in local T-cell regulation, although direct functional evidence in HCC remains limited (23, 24).

Notably, recent spatial multi-omics studies have identified a CCL19^+^ fibroblast state associated with T-cell infiltration and immunotherapy benefit, further suggesting that the immunological effects of CAFs in HCC are not uniformly suppressive but instead vary according to subset identity and spatial context (25). However, these transcriptionally defined CAF states cannot be simply equated with the classical myCAF, iCAF, or apCAF categories, and their lineage origins have not all been demonstrated to derive from HSCs. Accordingly, findings related to transcriptionally defined or spatially resolved CAF populations should not automatically be attributed to HSC-derived CAFs. Discussions of HSC-derived CAF–T-cell interactions therefore need to distinguish CAF cellular origin, functional state, and spatial localization.

As liver-specific stromal cells, quiescent HSCs are mainly located in the perisinusoidal space, where they participate in vitamin A storage and the maintenance of tissue homeostasis (26). Under sustained stimulation by chronic inflammation and tumor-associated signals, HSCs can transition into myofibroblast-like cells, some of which may further acquire CAF-like features (27, 28). HSC-derived CAFs are generally characterized by strong matrix-producing and tissue-remodeling capacities and may express molecules such as α-SMA, FAP, PDGFR, and COL1A1 while contributing to the deposition of ECM components including collagen, fibronectin, and hyaluronic acid (29–31). However, these markers are not uniquely specific to HSC-derived CAFs and may also be expressed by other activated fibroblast populations. Thus, within the broader context of CAF heterogeneity, HSC-derived CAFs are particularly relevant to fibrotic matrix formation and tissue stiffness, whereas the extent to which they specifically determine spatial T-cell restriction may vary according to lineage, functional state, and local stromal organization.

Compared with other solid tumors, HSC-derived CAF–T-cell interactions in HCC occur within a highly organ-specific hepatic microenvironment. The liver is continuously exposed to gut-derived antigens and microbial signals delivered through the portal circulation and must maintain a dynamic balance between immune surveillance and immune tolerance. Liver sinusoidal endothelial cells, Kupffer cells, HSCs, and intrahepatic lymphocytes collectively contribute to this homeostasis. HSCs reside in the space of Disse between liver sinusoidal endothelial cells and hepatocytes, enabling them to simultaneously sense hepatocyte injury, circulating signals, and local immune responses while contributing to perisinusoidal matrix deposition and spatial remodeling following chronic injury. Recent single-cell-resolution spatial transcriptomic studies of human liver tissue have further shown that fibrosis is accompanied by reorganization of the spatial architecture and cellular communication of HSCs, macrophages, and other non-parenchymal cells (32). Therefore, the transition of HSCs toward CAF-like states generally occurs within a multicellular tissue ecosystem in which chronic inflammation, fibrosis, and even cirrhosis are already established.

This organ-specific context is further shaped by HCC etiology and metabolic status. Persistent viral antigen stimulation in chronic viral hepatitis can promote dysfunction of virus-specific CD8^+^ T cells and remodeling of intrahepatic immunity, whereas metabolic stress in metabolic dysfunction-associated steatotic liver disease/metabolic dysfunction-associated steatohepatitis (MASLD/MASH) is accompanied by HSC activation, enhanced profibrotic programs, and reprogramming of the functional states of intrahepatic immune cells (33–35). Recent single-cell studies of the progression from MASH to HCC have further shown remodeling toward immunosuppressive and exhausted states among CD4^+^, CD8^+^, and γδ T cells during disease progression, suggesting that the metabolic liver disease background itself may precondition the immune ecosystem of subsequent HCC (36). As fibrosis progresses toward cirrhosis, the formation of fibrous septa, sinusoidal capillarization, redistribution of blood flow, and local hypoxia may further alter the spatial proximity and conditions for cellular communication between HSCs, CAFs, and T cells. Thus, CAF–T-cell crosstalk in HCC cannot be fully explained by classical CAF models derived from other solid tumors but should instead be understood as an immune–stromal network jointly shaped by chronic liver injury, intrinsic hepatic immune tolerance, metabolic abnormalities, and liver-specific spatial architecture. Within this network, HSC-derived CAFs represent an important stromal component, but not all CAF-related interactions can be assigned specifically to an HSC lineage.

T cells likewise exhibit marked subset and functional heterogeneity. CD8^+^ cytotoxic T cells mediate the principal tumor-killing response (37), whereas CD4^+^ helper T cells contribute to antitumor immunity through cytokine networks and cooperation with other immune cells (38, 39). In contrast, Treg accumulation can further enhance local immunosuppression (40, 41). Recent single-cell and spatial studies have identified a CD177^+^ tumor-infiltrating Treg subset with pronounced immunosuppressive features and have linked this population to HCC progression and immunotherapy resistance (42). In HCC, effector T cells frequently exhibit insufficient infiltration, abnormal spatial distribution, and functional exhaustion. Some CD8^+^ T cells are predominantly localized at the tumor margin, within fibrous septa, or around tumor-associated vessels and therefore fail to adequately penetrate the tumor parenchyma (43–45). Highly multiplexed spatial imaging studies have further demonstrated substantial interpatient heterogeneity in the spatial proximity between tumor cells and CD8^+^ T cells in HCC, with this proximity being associated with clinical outcomes (46). This spatial mismatch suggests that CAF-associated stromal remodeling may affect not only T-cell abundance but also their ability to establish effective contact with tumor cells. However, unless lineage tracing or other direct evidence is available, such spatial CAF effects should not be assumed to arise exclusively from HSC-derived CAFs.

Recent single-cell studies have also revealed substantial heterogeneity among γδ T cells in HCC, identifying IFN-γ-producing, IL-17-associated/cytotoxic, and antigen-presenting-like states, suggesting that different γδ T-cell subsets may exert distinct or even opposing immune functions (47). Mucosal-associated invariant T (MAIT) cells are relatively enriched in the human liver, whereas intratumoral MAIT cells in HCC may exhibit increased expression of inhibitory molecules such as PD-1, CTLA-4, and TIM-3, reduced cytotoxic function, and protumor functional reprogramming (48). Tissue-resident memory T (TRM) cells also exhibit substantial subset heterogeneity. Recent single-cell RNA sequencing and single-cell TCR sequencing studies in HCC have identified CD69^+^ and CD103^+^ tumor-associated TRM subsets and demonstrated differences in their transcriptional programs, clonal architecture, developmental trajectories, and recurrence-associated cell–cell communication (49). Direct causal evidence linking these unconventional or tissue-resident T-cell subsets specifically to HSC-derived CAFs remains limited. Whether they are selectively regulated by HSC-derived CAFs, other CAF subsets, stromal structures, or multicellular signaling networks therefore requires further validation.

In addition, CD8^+^ T-cell exhaustion is not a single terminal state but instead encompasses a continuum of states with different proliferative capacities and therapeutic responsiveness. Recent single-cell studies in HCC have further confirmed pronounced heterogeneity in intratumoral CD8^+^ T-cell exhaustion and linked specific exhaustion states to responses to immunotherapy (50). Of particular note, a 2026 HCC study further constructed an atlas of exhausted CD8^+^ T cells, showing that progenitor-exhausted T cells (Tpex) retain a degree of stemness and proliferative potential, whereas progression toward terminal exhaustion is accompanied by sustained functional decline. The balance between FYN and LCK and the associated metabolic reprogramming were implicated in this differentiation process and in the response to anti-PD-1 therapy (51). Therefore, the effects of CAF-derived or CAF-associated signals, including TGF-β, CXCL12, inflammatory mediators, and ECM barriers, may not be uniform across different T-cell subsets and exhaustion states. Moreover, because several of these mediators can originate from multiple cellular populations, their effects should not be attributed specifically to HSC-derived CAFs unless supported by lineage-resolved evidence. Their specific roles need to be resolved in the context of CAF origin, functional state, T-cell subset, spatial localization, and disease stage. Overall, both CAFs and T cells should be regarded as heterogeneous populations defined by distinct origins, states, and spatial distributions, and this dual heterogeneity constitutes the cellular basis for understanding bidirectional crosstalk between HSC-derived CAFs and T cells in HCC.

## 3 Bidirectional crosstalk between HSC-derived CAFs and T cells

### 3.1 Regulation of T cells by HSC-derived CAFs

**Figure 1:**
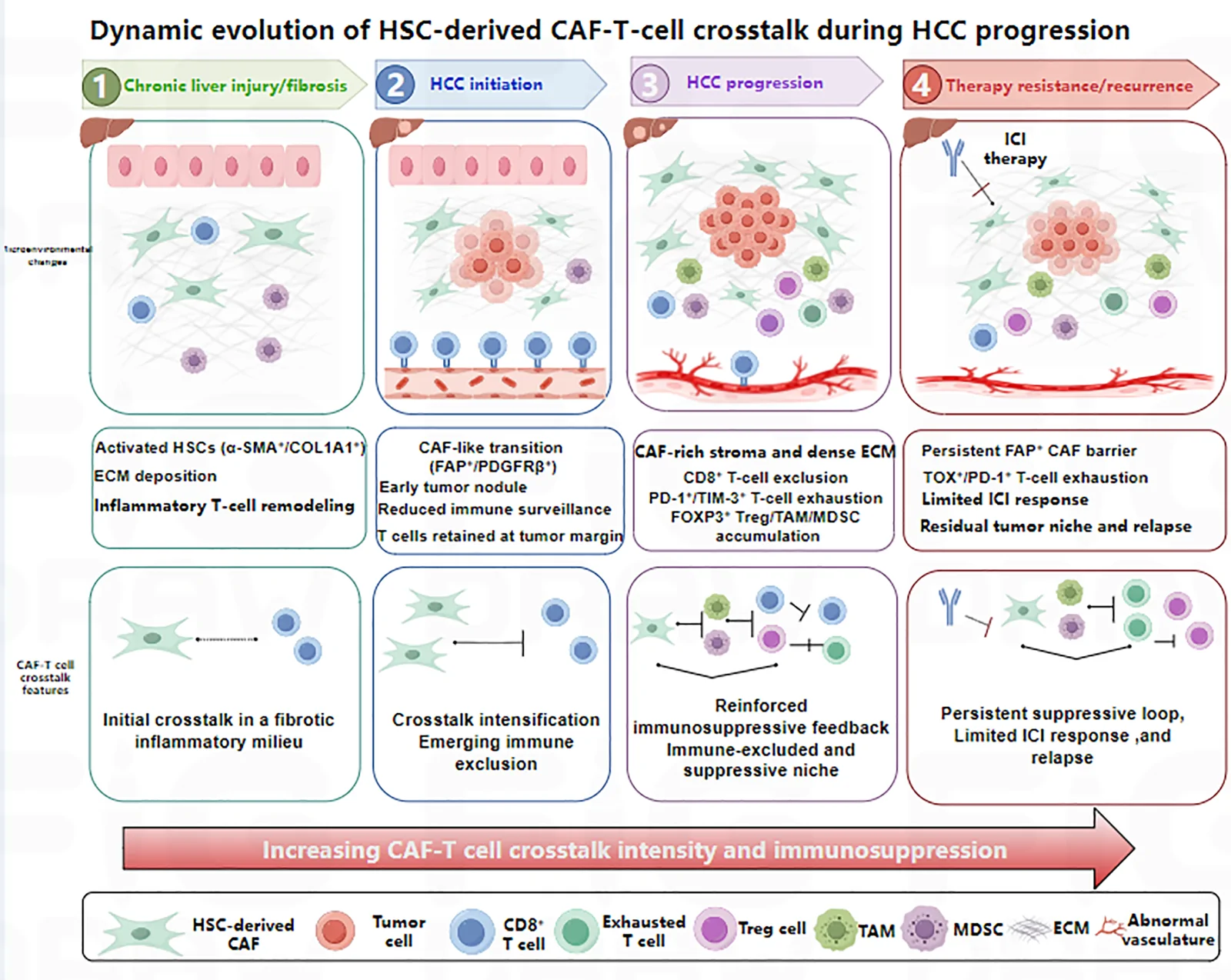
Major mechanisms of HSC-derived CAF–mediated T-cell dysfunction and immune escape in HCC. This schematic summarizes the major mechanisms through which HSC-derived CAFs and associated stromal programs may restrict antitumor T-cell responses in HCC. First, immunosuppressive cytokine signaling, represented by TGF-β/SMAD2/3 and IL-6/STAT3, can impair CD8^+^ T-cell cytotoxicity and function while favoring Treg accumulation. Second, CXCL12/CXCR4-mediated chemotactic signaling can alter effector T-cell trafficking and promote their retention at stromal or peritumoral regions, thereby restricting entry into the tumor core. Third, CCL2/CCR2-associated myeloid-cell recruitment and related HSC–myeloid regulatory networks can promote the accumulation of TAMs and MDSCs, indirectly suppressing CD8^+^ T-cell activity. In parallel, ECM remodeling, VEGF-related vascular abnormalities, and hypoxia create physical and vascular barriers that further impair T-cell infiltration. Collectively, these interconnected mechanisms contribute to T-cell exclusion, immune escape, and limited therapeutic response in HCC. Unless otherwise specified, the cytokines, chemokines, and other mediators depicted in this image should not be interpreted as being exclusively derived from HSC-derived CAFs. Several signals, including IL-6, CCL2, and VEGF, may originate from immune cells, endothelial cells, hepatocytes, and other stromal populations within the HCC microenvironment.

The regulation of T cells by HSC-derived CAFs involves multiple levels, including immunosuppressive signaling, alterations in chemokine networks, amplification of inflammatory immunosuppression, indirect regulation through myeloid cells, and the formation of ECM barriers. Within the broader CAF ecosystem, HSC-derived CAFs may contribute to restricting the entry of effector T cells into the tumor parenchyma, impairing CD8^+^ T-cell cytotoxic function, promoting the accumulation of immunosuppressive cells such as Tregs, and supporting the establishment of an immune-excluded or immune-cold microenvironment in HCC. **Figure 1** summarizes the major HSC-derived CAF-associated and multicellular mechanisms implicated in T-cell dysfunction and immune evasion, including TGF-β/SMAD- and IL-6/STAT3-related immunosuppressive signaling, CXCL12/CXCR4-mediated spatial exclusion, CCL2/CCR2-associated myeloid regulation, and stromal–vascular barriers formed through ECM remodeling, abnormal vasculature, and hypoxia. **Table 1** further summarizes these mechanisms in terms of CAF states, molecular pathways, targeted immune components, mechanistic interpretations, strength of evidence, translational implications, and key limitations.

**Table 1:** Major mechanisms by which HSC-derived CAFs regulate T-cell function in HCC.

Regulatory level	CAF subset/phenotype	Molecular pathway	Targeted T-cell or immune component	Mechanistic interpretation	Experimental evidence and strength	Translational implication	Key limitations	References
Immunosuppressive signaling	Activated HSC-derived CAFs/myCAF-like phenotype; specific HSC-derived subset not definitively resolved	TGF-β1–TGFBR2/TGFBR1–SMAD2/3–SMAD4	CD8^+^ T cells, Tregs	TGF-β activates SMAD2/3–SMAD4 transcriptional programs that sustain HSC/CAF activation and ECM production, while simultaneously suppressing CD8^+^ T-cell effector function and favoring immune tolerance and Treg accumulation	Strong evidence for TGF-β/SMAD-mediated HSC activation and fibrosis; substantial tumor and immune evidence; direct human HCC evidence linking a defined HSC-derived CAF subset to specific T-cell states remains limited	Targeting the TGF-β/TGFBR axis may help relieve stromal immunosuppression and improve T-cell function, particularly when combined with immune checkpoint blockade; however, patient selection and stromal context are likely to influence therapeutic benefit.	TGF-β has pleiotropic and multicellular effects; systemic blockade may impair liver regeneration, tissue repair, and immune homeostasis, while HSC-derived CAF-specific targeting remains unavailable.	(52–55)
Chemokine-mediated regulation	Peritumoral apCAF-like CAFs; HSC lineage origin not definitively established	CXCL12–CXCR4–Gαi/PI3K–AKT/ERK chemotactic signaling	CD8^+^ effector T-cell trafficking and spatial distribution	CXCL12–CXCR4 signaling alters T-cell chemotaxis and spatial retention, favoring accumulation at stromal or tumor-margin regions and restricting effective entry into the tumor parenchyma	Moderate evidence from human HCC spatial transcriptomics and tissue analyses supporting spatial association with CD8^+^ T-cell exclusion; direct subset-specific functional causality remains incomplete	Potential strategy for converting immune-excluded tumors into more permissive immune states; therapeutic benefit may depend on CAF subtype, spatial localization, and baseline T-cell distribution	CXCL12 is produced by multiple stromal and immune populations; HSC-lineage specificity remains unresolved, and systemic targeting may disrupt physiological lymphocyte trafficking.	(23, 56–58)
Inflammatory immunosuppression	iCAF-like inflammatory phenotype; HSC lineage origin not definitively established	IL-6–IL-6R/gp130–JAK1/2–STAT3 (Tyr705)–c-MAF	CD8^+^ T cells, Tregs, local inflammatory networks	Persistent IL-6 signaling activates STAT3-dependent transcriptional programs and promotes CD8^+^ T-cell dysfunction/exhaustion, including increased expression of inhibitory receptors, while reinforcing an inflammatory immunosuppressive microenvironment	Moderate-to-strongevidence from HCC patient samples and functional studies for IL-6/STAT3-mediated T-cell dysfunction; CAF-specific IL-6 origin has not been definitively established	Provides a rationale for combining IL-6/STAT3 inhibition with ICIs, although the lack of CAF-source specificity may limit biomarker selection and increase off-target effects	IL-6 has diverse cellular sources and cannot be attributed specifically to HSC-derived CAFs; current strategies do not enable CAF-specific IL-6 blockade and may produce broad immunological effects.	(59–62)
Myeloid-cell recruitment and reprogramming	Activated α-SMA^+^ HSCs/HSC-derived CAF-like cells	CCL2–CCR2; CX3CL1–CX3CR1–retinoid–ARG1 axis	Monocytes, TAMs/MDSCs, and indirectly CD8^+^ T cells	CCL2–CCR2 promotes recruitment of suppressive myeloid cells, whereas activated HSC-derived signals can further reprogram CX3CR1^+^ macrophages through retinoid-dependent ARG1 induction, resulting in arginine depletion and impaired CD8^+^ T-cell proliferation and function	Strong multimodal evidence for HSC–macrophage–CD8^+^ T-cell regulation, including human HCC single-cell and tissue analyses, *in vitro* coculture, adoptive-transfer experiments, and genetically or pharmacologically manipulated mouse models; CCL2 itself is not CAF-specific	Supports combined stromal–myeloid targeting; however, chemokine redundancy and alternative myeloid-recruitment pathways may limit the durability of single-axis blockade	CCL2 arises from multiple cellular sources, and pathway redundancy or compensatory myeloid-recruitment mechanisms may limit the durability of single-axis blockade.	(63–66)
Stromal barrier	FAP^+^ CAFs/ECM-producing myCAF-like CAFs	COL1A1/FN1-rich ECM, collagen organization, matrix stiffness, and mechanotransduction	T-cell infiltration and migration	Excessive collagen and fibronectin deposition increases stromal density and stiffness, creating a physical and mechanical barrier that restricts effector T-cell migration and access to tumor nests; FAP^+^ CAF-rich niches are spatially associated with immune exclusion	Moderate-to-strongevidence from human HCC single-cell and spatial transcriptomics, multiplex immunofluorescence/spatial analyses, patient cohorts, and preclinical FAP-directed intervention studies	Matrix normalization or selective FAP^+^ CAF modulation may improve T-cell penetration and ICI responsiveness, but excessive stromal depletion could impair tissue integrity or remove potentially tumor-restraining CAF populations	FAP, α-SMA, and related markers are not HSC-lineage specific; excessive stromal depletion may compromise tissue integrity or eliminate potentially protective CAF states.	(21, 24, 67–69)
Vascular and hypoxia-related regulation	VEGFA^+^ CAFs, predominantly tumor-interior; HSC lineage origin not definitively established	Hypoxia/HIF-1α–VEGFA–VEGFR2 signaling	Endothelial cells, T-cell infiltration, immune-cell recruitment	Hypoxia promotes a VEGFA-high CAF state that enhances abnormal angiogenesis and vascular organization, indirectly reducing effective immune-cell trafficking and reinforcing a hypoxic, immunosuppressive microenvironment	Moderate evidence from human HCC single-cell and spatial transcriptomics, ligand–receptor analyses, and clinical outcome/therapeutic-response associations; direct causal effects on T cells remain less well established	Provides a mechanistic rationale for stromal/antiangiogenic–ICI combinations, but therapeutic efficacy is likely to depend on vascular state, CAF spatial distribution, and underlying liver disease	VEGF is not CAF specific and is produced by multiple cell types; vascular targeting may affect normal hepatic vasculature, and direct T-cell effects remain incompletely defined.	(22, 70–72)

At the level of immunosuppressive signaling, the TGF-β/SMAD axis represents an important pathway through which CAF populations, including HSC-derived CAFs, may influence T-cell function. CAF-derived TGF-β can impair the cytotoxic activity of CD8^+^ T cells and promote the accumulation of immunosuppressive T-cell subsets such as Tregs, thereby reducing the intensity of local antitumor immune responses. In contrast, the CXCL12/CXCR4 axis primarily affects the spatial distribution of T cells. CAF-associated CXCL12 signaling can alter the migration and localization of effector T cells, preventing their efficient entry into the tumor parenchyma and thereby contributing to the formation of a T-cell-excluded microenvironment. However, neither TGF-β nor CXCL12 should be considered uniquely specific to HSC-derived CAFs, because both may be produced by multiple stromal and non-stromal cell populations within the HCC microenvironment.

At the level of inflammation-associated immunosuppression, IL-6/STAT3 signaling can sustain chronic inflammatory and immunosuppressive states, leading to progressive functional decline of T cells under persistent stimulation. Likewise, CCL2/CCR2-related signaling can recruit monocytes, tumor-associated macrophages, and myeloid-derived suppressor cells, which can further release immunosuppressive mediators and indirectly restrict CD8^+^ T-cell activation and cytotoxic function. CAFs, including HSC-derived CAF-like populations, may contribute to these signaling networks; however, IL-6 and CCL2 originate from multiple cellular sources and should therefore be interpreted as components of a multicellular immunoregulatory network rather than as HSC-derived CAF-specific mediators. Through these indirect interactions with myeloid populations, CAF-associated stromal programs may further weaken T-cell responses and reinforce local immune suppression.

In addition to soluble signals, HSC-derived CAFs may contribute to T-cell exclusion through ECM deposition and stromal remodeling. Accumulation of matrix components such as collagen, fibronectin, and hyaluronic acid can increase tissue stiffness and create spatial barriers that restrict T-cell migration. Abnormal angiogenesis and a hypoxic microenvironment may further reduce effective T-cell infiltration while reinforcing local immunosuppression. Although HSC-derived CAFs are particularly relevant to fibrotic matrix production in the liver, ECM remodeling and vascular abnormalities represent multicellular processes involving other fibroblast subsets, endothelial cells, tumor cells, and immune populations. Their effects on T-cell localization should therefore not be attributed exclusively to an HSC lineage.

CAF-associated metabolic reprogramming may also influence T-cell function. Activated CAFs in the tumor microenvironment exhibit high metabolic activity and can alter the local allocation of metabolic resources through competitive consumption of glucose, glutamine, and other essential nutrients, thereby restricting T-cell proliferation, survival, and effector function. This may be particularly relevant for CD8^+^ T cells, which rely on glycolysis and glutamine metabolism to sustain cytotoxic activity. CAF-mediated nutrient competition may therefore increase metabolic stress in CD8^+^ T cells and promote dysfunction and exhaustion. However, the extent to which these metabolic effects are specifically mediated by HSC-derived CAFs in HCC remains incompletely defined.

CAF-derived metabolites may also participate in immune regulation. Increased glycolysis in CAFs can result in lactate accumulation and acidification of the tumor microenvironment, suppressing the cytotoxic function of CD8^+^ T cells and promoting the maintenance of immunosuppressive states. In parallel, aberrant activation of adenosine metabolic pathways may reduce T-cell activation through adenosine receptor-related signaling. CAF-mediated lipid metabolic remodeling may also affect T-cell membrane composition, signal transduction, and metabolic adaptability, thereby further altering antitumor function. In recent years, increasing attention has been paid to the relationships between CAF-mediated regulation of oxidative stress, lipid metabolism, and iron homeostasis and the susceptibility of tumor cells to ferroptosis. However, direct evidence that HSC-derived CAFs regulate T-cell function through these metabolic or ferroptosis-related networks in HCC remains limited, and most such mechanisms should currently be regarded as CAF-associated or stromal hypotheses rather than established HSC-lineage-specific pathways.

Importantly, the mechanisms described above are unlikely to operate independently but instead may constitute an interconnected immunosuppressive network. CAF-derived TGF-β signaling can directly suppress CD8^+^ T-cell effector function while simultaneously promoting fibroblast activation and ECM deposition, thereby further reinforcing spatial T-cell exclusion. CXCL12/CXCR4-mediated chemotactic abnormalities and stromal barriers may act together to restrict the entry of effector T cells into the tumor parenchyma, whereas inflammatory and myeloid regulatory pathways such as IL-6/STAT3 and CCL2/CCR2 may sustain local immunosuppression on this basis. Hypoxia and metabolic reprogramming may further amplify these processes, exposing T cells simultaneously to spatial restriction, nutrient deprivation, and persistent inhibitory signals. Therefore, the regulation of T cells by HSC-derived CAFs is best understood within a broader stromal–immune network characterized by functional redundancy and synergistic effects. Current evidence suggests that stromal remodeling and TGF-β-related signaling may constitute a relatively fundamental structural and functional framework, whereas chemotactic, inflammatory, myeloid, and metabolic mechanisms may further reinforce and maintain the immunosuppressive state. The strength of evidence nevertheless differs substantially across these mechanisms. Some are supported by HCC tissue, functional, or lineage-related studies, whereas others are derived primarily from general CAF studies, preclinical models, or mechanistic extrapolation from other tumor types. Their direct causal roles in HSC-derived CAF–T-cell crosstalk therefore require further validation.

### 3.2 Reverse remodeling of HSC-derived CAFs by T cells

Within the HCC microenvironment, T cells are not merely passive targets of stromal regulation. Cytokines released by T cells can in turn influence CAF activation states, inflammatory phenotypes, and matrix-remodeling capacity. IL-17A is one of the key signals linking T-cell-mediated inflammation to fibroblast activation (73). Th17 cell-derived IL-17A can engage the IL-17 receptor complex on CAFs or activated fibroblast-like cells and activate NF-κB and MAPK signaling through ACT1/TRAF6 (74), thereby promoting the release of inflammatory mediators and chemokines such as IL-6, CXCL8, and CCL2 while enhancing the expression of activation- and matrix-associated molecules including α-SMA and COL1A1 (75–77). Similarly, sustained TNF-α stimulation can enhance the production of inflammatory mediators and chemokines by CAFs through TNFR-associated NF-κB and MAPK signaling (15, 78). However, direct evidence demonstrating that these effects selectively remodel HSC-derived CAFs in human HCC remains limited.

TGF-β is primarily involved in maintaining fibroblast activation and matrix-remodeling phenotypes. Local Tregs in HCC can serve as an important source of TGF-β (79). Upon binding to TGF-β receptors on CAFs or activated HSC/myofibroblast-like cells, TGF-β activates SMAD2/3 signaling and promotes the formation of transcriptional regulatory complexes with SMAD4, thereby increasing the expression of α-SMA, COL1A1, fibronectin, and other matrix-associated molecules and maintaining a myofibroblast-like, matrix-producing state (29, 80, 81). Because TGF-β also exerts direct immunosuppressive effects, sustained TGF-β signaling may further reinforce CAF-associated spatial T-cell exclusion and immune tolerance (82, 83). Compared with other reverse regulatory pathways, the experimental basis for TGF-β–SMAD-mediated HSC activation and fibrosis is relatively well established. However, direct causal evidence in human HCC demonstrating that Treg-derived TGF-β continuously shapes specific HSC-derived CAF subsets remains insufficient. Its ability to act simultaneously on stromal and immune compartments makes this pathway highly attractive from a translational perspective, whereas its broad involvement in liver homeostasis and tissue repair limits the safety window for nonselective systemic blockade.

In addition to proinflammatory and profibrotic signals, immunoregulatory cytokines derived from T cells may also shape CAF function. IL-10 released by Tregs or other immunoregulatory T-cell populations can influence the immunoregulatory state of fibroblasts through IL-10R-associated JAK/STAT signaling and may contribute to the maintenance of a locally hyporesponsive immune environment (84–86). In contrast, IFN-γ produced by Th1 cells and activated CD8^+^ T cells displays more pronounced context dependence. During effective antitumor immunity, IFN-γ generally enhances antigen presentation and immune recognition (87); however, under conditions of chronic inflammation and persistent antigenic stimulation, sustained IFN-γ signaling may also induce feedback immunoregulatory effects (88). Overall, evidence that IL-10 or IFN-γ directly remodels HSC-derived CAFs in HCC remains limited, and many current interpretations are still derived from fibrosis studies, general fibroblast biology, other tumor types, or cell–cell communication inference.

Taken together, current evidence suggests that HSC-derived CAF–T-cell crosstalk is best understood as a bidirectional immune–stromal regulatory network that dynamically evolves with disease progression rather than as a set of isolated, lineage-specific signaling pathways. During chronic liver injury and fibrosis, persistent inflammation initially promotes HSC activation and T-cell functional remodeling, generating a protumor microenvironment with profibrotic and immune-tolerant features. As HCC develops, a proportion of activated HSCs may acquire CAF-like characteristics and participate in matrix deposition, chemotactic dysregulation, and immunoregulatory signaling that impair T-cell immune surveillance. During tumor progression, broader CAF subset heterogeneity, spatial barriers, immunosuppressive signals, and metabolic stress increasingly reinforce T-cell exclusion and exhaustion, gradually establishing a stable immunosuppressive ecosystem. Under therapeutic pressure, CAF plasticity, compensatory immunosuppressive pathways, and persistent stromal architecture may further limit the restoration of T-cell function following checkpoint blockade and contribute to therapeutic resistance and the formation of residual tumor niches.

Importantly, this process is neither unidirectional nor a fixed linear progression. Different CAF subsets may exert protumor, immunosuppressive, tissue-restraining, or potentially immune-supportive functions, while different T-cell subsets may promote HSC/CAF activation under some conditions and participate in the elimination of activated HSCs under others. Signals such as IFN-γ likewise exhibit marked context dependence. The ultimate effects of CAF–T-cell crosstalk may therefore be determined by the combined influence of fibroblast lineage, functional state, T-cell subset composition, spatial localization, disease stage, and therapeutic pressure. This stage-dependent bidirectional feedback model provides the integrative conceptual framework used in this review to understand immune evasion and therapeutic resistance in HCC while avoiding over-attribution of general CAF biology to HSC-derived CAFs.

## 4 Dynamic roles of HSC-derived CAF–T cell crosstalk in HCC development and therapeutic resistance

**Figure 2:**
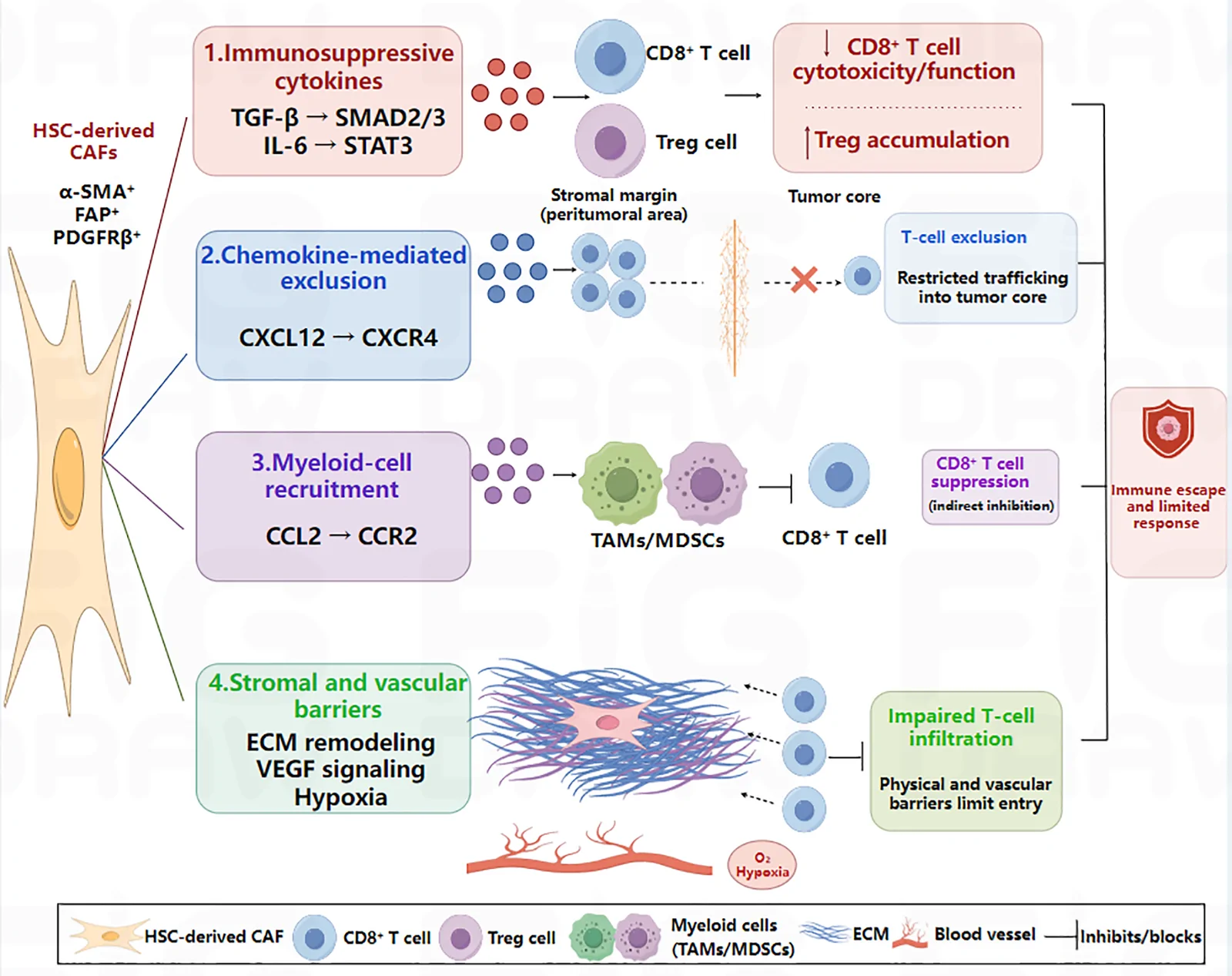
Stage-dependent evolution of HSC-derived CAF–T-cell crosstalk during HCC progression and therapy resistance. This image presents a conceptual framework for the stage-dependent evolution of HSC-derived CAF–T-cell crosstalk from chronic liver injury and fibrosis to HCC progression, therapy resistance, and recurrence. During chronic liver injury and fibrosis, activated HSCs characterized by α-SMA and COL1A1 expression, together with ECM deposition and inflammatory T-cell remodeling, establish a fibrotic and immune-altered premalignant environment. During HCC initiation, a subset of activated HSCs may acquire CAF-like features, including FAP and PDGFRβ expression, while reduced immune surveillance and retention of T cells at the tumor margin favor early immune escape. As HCC progresses, CAF-rich stroma and dense ECM increasingly coincide with CD8^+^ T-cell exclusion, PD-1^+^/TIM-3^+^ T-cell exhaustion, and accumulation of Tregs, TAMs, and MDSCs, reinforcing an immune-excluded and immunosuppressive niche. Under therapeutic pressure, persistent FAP^+^ CAF-associated stromal barriers, TOX^+^/PD-1^+^ exhausted T-cell states, and suppressive cellular networks may limit T-cell reinvigoration after immune checkpoint blockade and contribute to residual tumor niches, therapeutic resistance, and recurrence. The increasing crosstalk intensity shown in the image represents an integrative working model based on evidence of differing strengths and should not be interpreted as a universally fixed or fully validated linear sequence. Unless otherwise specified, the cytokines, chemokines, and other mediators depicted in this image should not be interpreted as being exclusively derived from HSC-derived CAFs. Several signals, including IL-6, CCL2, and VEGF, may originate from immune cells, endothelial cells, hepatocytes, and other stromal populations within the HCC microenvironment.

Bidirectional interactions between HSCs/HSC-derived CAF-like cells and T cells may extend across multiple stages of disease, from chronic liver injury and fibrosis to HCC initiation, tumor progression, and therapeutic resistance. Examining CAF–T-cell crosstalk from a disease-stage perspective may help explain how early inflammatory–fibrotic responses gradually evolve into stromal and immune conditions that favor immune evasion and treatment failure in HCC. **Figure 2** summarizes the dynamic changes in HSC activation, CAF-associated stromal programs, T-cell states, and immune organization across different disease stages.

It should be emphasized that this stage-based framework is intended to integrate available mechanistic and temporal evidence and does not imply that each stage has been validated with the same degree of lineage-specific or causal evidence. HSC–T-cell interactions during chronic liver injury and fibrosis are supported by a relatively substantial body of evidence from animal models and fibrotic tissue studies, whereas associations between CAF enrichment, stromal activation, T-cell exclusion, and exhaustion during established HCC are increasingly supported by single-cell analyses, spatial omics, and patient tissue studies. By contrast, direct causal relationships between HSC-derived CAFs and specific T-cell subsets during early HCC development, therapeutic resistance, and recurrence remain limited. Some stage-specific interpretations therefore rely on preclinical models, omics-based correlations, general CAF biology, or mechanistic extrapolation from other solid tumors. The descriptions below should accordingly be regarded as a working model rather than as a fully validated lineage-resolved trajectory of disease progression.

### 4.1 Chronic liver injury and fibrosis: establishing the premalignant soil for subsequent CAF–T-cell crosstalk

Most HCCs arise in the setting of chronic liver disease and fibrosis. During chronic hepatitis, the liver is persistently exposed to inflammatory stimuli, oxidative stress, and signals associated with metabolic abnormalities, which repeatedly activate HSCs and gradually drive their transition toward myofibroblast-like states (29). At this stage, a typical tumor microenvironment has not yet formed; however, HSC activation, extracellular matrix deposition, and persistent immune-cell infiltration already establish a stromal and immunological context that may facilitate the subsequent emergence of HSC-derived CAF-like populations and HCC-associated immune escape.

Animal studies suggest that HSC activation during chronic liver injury and fibrosis is frequently accompanied by remodeling of T-cell subsets (89). In models of metabolic liver injury, Th17-associated inflammatory signaling can promote HSC activation and collagen deposition, whereas increased Treg activity may limit inflammatory tissue damage while simultaneously favoring a more immune-tolerant local environment (90). In addition, some models of fibrosis regression indicate that specific CD8^+^ T-cell subsets may participate in the clearance of activated HSCs, suggesting that T-cell–HSC interactions are stage dependent and are not uniformly profibrotic (91).

Omics studies further support the presence of a complex immune–stromal ecosystem in fibrotic liver tissue. Single-cell analyses have shown that HSCs and myofibroblast-like stromal populations can acquire matrix-producing, inflammatory, and immunoregulatory transcriptional programs during fibrosis (92, 93). At the same time, intrahepatic T cells may display transcriptional features associated with chronic antigen stimulation, clonal expansion, exhaustion, or immune regulation (94). Studies of human cirrhotic liver tissue have also revealed extensive cellular communication among stromal cells, T cells, macrophages, and endothelial cells within fibrotic lesions (95). Together, these findings provide a cellular basis for the later transition from HSC–T-cell interactions in chronic liver disease to broader CAF–T-cell crosstalk in established HCC.

Chronic liver injury and fibrosis may therefore be regarded as a premalignant soil in which stromal remodeling and immune adaptation precede the development of overt HCC. During this stage, HSCs progressively acquire matrix-remodeling and immunoregulatory properties, whereas T cells undergo functional remodeling under persistent inflammatory and antigenic stimulation. The resulting inflammatory–fibrotic–immune-tolerant environment may create conditions that facilitate the survival of abnormal hepatocytes and their subsequent escape from immune surveillance. Importantly, because most evidence at this stage concerns activated HSCs, myofibroblast-like cells, and fibrotic stromal populations rather than lineage-resolved HSC-derived CAFs, these early interactions should be distinguished from the more established CAF–T-cell networks observed after HCC development.

### 4.2 HCC initiation: impaired immune surveillance and the emergence of early immune escape

Following the establishment of an inflammatory–fibrotic microenvironment, a subset of abnormal hepatocytes may accumulate additional genetic and epigenetic alterations and gradually evolve into early tumor clones (96). Unlike the chronic fibrosis stage, the central issue during HCC initiation is no longer limited to HSC activation and dysregulated tissue repair but increasingly concerns whether abnormal hepatocytes can be efficiently recognized and eliminated by the immune system. When stromal remodeling, impaired antigen presentation, and restricted T-cell effector function coexist, early tumor cells are more likely to evade immune surveillance, thereby creating an immunological environment that favors tumor initiation (97, 98).

During this process, activated HSCs and HSC-derived CAF-like cells may contribute to local stromal remodeling and thereby influence T-cell access to emerging tumor cells (99). However, direct evidence demonstrating that these early stromal effects are mediated specifically by HSC-lineage CAFs remains limited. The stromal environment associated with early HCC gradually acquires features that favor the survival of emerging tumor clones, including restricted entry of effector T cells, increased accumulation of immunoregulatory cells, and enhanced immune-tolerant signaling (100–102). Animal studies have further shown that inhibition of HSC activation or attenuation of profibrotic signaling can reduce tumor burden and improve local immune infiltration (103), supporting a role for the fibrotic stromal context in shaping early immune escape.

Single-cell sequencing has further suggested that local remodeling among stromal cells, immune cells, and abnormal hepatocytes occurs in early HCC and adjacent non-tumor regions (104). CAF-like populations may display matrix-producing and immunoregulatory features, whereas T cells may exhibit reduced effector function, increased expression of checkpoint molecules, or an increased proportion of Tregs (12, 105). Spatially, CAF-enriched regions are often accompanied by restricted or marginalized distributions of effector T cells, whereas immunosuppressive cells are more likely to accumulate in stromal-rich areas (106, 107). This spatial mismatch may reduce the likelihood that early tumor cells are effectively targeted by T cells. However, these CAF-enriched spatial patterns do not by themselves establish an HSC lineage for the fibroblast populations involved.

CAF–T-cell crosstalk during HCC initiation may therefore be characterized by impaired immune surveillance and the emergence of early immune escape. Activated HSCs and HSC-derived CAF-like cells may participate in this process through matrix remodeling and immunoregulatory effects, while broader CAF and stromal populations can also contribute to altered T-cell entry, localization, and function. Reduced T-cell clearance capacity in turn creates opportunities for the expansion of abnormal hepatocytes. Direct lineage-resolved evidence defining the relative contribution of HSC-derived CAFs at this stage remains an important unmet need.

### 4.3 HCC progression: CAF barriers and T-cell exhaustion stabilize immune escape

As HCC progresses, abnormal CAF–T-cell interactions established at earlier stages may shift from a relatively dynamic immune imbalance toward a more stable immunosuppressive ecosystem. Compared with the tumor-initiation stage, a more prominent feature of established HCC is the persistent coexistence of matrix-producing, inflammation-regulating, and immunosuppression-associated CAF states with exhausted CD8^+^ T cells, Tregs, and immunosuppressive myeloid cells, together forming a spatially organized immune-evasion network (43, 108, 109).

Single-cell, spatial-omics, and patient tissue studies have shown that CAF enrichment and stromal activation in advanced HCC are often accompanied by marginalization of effector T cells, increased abundance of exhausted T cells, and accumulation of immunosuppressive myeloid cells (110, 111). Higher expression of CAF-associated markers or greater stromal enrichment scores are also generally associated with stronger immunosuppressive features and poorer prognosis (12, 112–114). These findings support an important role for CAF-associated stromal programs in advanced HCC but do not necessarily establish that the responsible CAF populations are uniformly HSC-derived.

Thus, the key change at this stage can be understood as the gradual transformation of CAF-associated stromal and immune programs from early localized abnormalities into a more stable, spatially organized, and self-reinforcing immunosuppressive ecosystem. HSC-derived CAFs may represent an important component of this process, particularly in tumors arising in fibrotic or cirrhotic livers, but their relative contribution is likely to depend on fibroblast lineage, functional state, and spatial localization. This evolving stromal–immune ecosystem provides a foundation for sustained tumor growth, invasion, T-cell dysfunction, and subsequent therapeutic resistance.

### 4.4 Therapeutic resistance and recurrence: CAF–T-cell feedback loops restrict immunotherapy responses

Under therapeutic pressure, CAF–T-cell crosstalk may contribute to the persistence of residual immunosuppressive niches. Even when checkpoints such as PD-1/PD-L1 are blocked, pre-existing stromal constraints, compensatory immunosuppressive pathways, and CAF plasticity may still prevent full restoration of effective antitumor T-cell activity (9, 115–118). At the same time, therapeutic pressure may select for tumor cell populations capable of adapting to immune attack and may promote reorganization of CAF states and local intercellular communication, together creating protective niches that favor the survival of residual tumor cells (119).

Animal experiments and translational studies suggest that CAF modulation, stromal remodeling, or blockade of related immunosuppressive pathways can enhance the antitumor effects of immune checkpoint inhibitors, whereas CAF enrichment and T-cell-excluded spatial features in patient samples are also associated with poorer therapeutic responses (120–123). However, most of these findings concern CAF-associated or stromal mechanisms broadly and do not establish that therapeutic resistance is driven specifically by HSC-derived CAFs. Likewise, treatment-induced changes in CAF states may reflect plasticity across multiple fibroblast lineages rather than selective remodeling of HSC-derived populations.

Accordingly, this stage places greater emphasis on the persistence, plasticity, and compensatory capacity of the broader CAF–T-cell network under therapeutic pressure and on its potential roles in primary resistance, acquired resistance, and recurrence. HSC-derived CAFs may contribute to this network, particularly in fibrotic HCC, but defining their lineage-specific role will require longitudinal and spatially resolved studies capable of distinguishing HSC-derived CAFs from other fibroblast populations before, during, and after therapy.

## 5 Clinical relevance and translational value of HSC-derived CAF–T-cell crosstalk

Although direct causal evidence for HSC-derived CAF–T-cell crosstalk still comes primarily from preclinical models and mechanistic studies, an increasing number of studies using HCC patient tissues and clinical cohorts have shown that CAF-related gene signatures and the degree of stromal activation are associated with tumor aggressiveness, immunosuppression, and adverse clinical outcomes (124, 125). More importantly, the clinical significance of CAFs does not depend solely on their overall abundance. Multi-omics and spatial studies have shown that CAF enrichment is often accompanied by marginalization of CD8^+^ T cells, accumulation of immunosuppressive cells, and immune exclusion, whereas the spatial distance between CAFs and T cells, their regional distribution, and the composition of local cellular niches may reflect the immune status of the HCC microenvironment more accurately than cell abundance alone (126, 127). However, most of these clinical and spatial observations concern CAF populations broadly and do not establish that the relevant fibroblast states are specifically derived from HSCs.

In recent years, single-cell and spatial transcriptomic studies have further revealed the pronounced spatial heterogeneity of the stromal–immune ecosystem in HCC. Different tumor regions display substantial differences in cellular composition, immune states, and intercellular communication, while distinct CAF states exhibit specific spatial localizations and functional programs. Stromal–immune signals such as TGF-β may also show regionally activated patterns at the tumor–stroma interface (128–131). These findings indicate that CAF subset identity, spatial localization, and proximity to T cells and other microenvironmental cells represent important dimensions for understanding immune exclusion in HCC and that CAF function cannot be inferred solely from overall CAF abundance. At the same time, transcriptionally or spatially defined CAF states should not automatically be interpreted as HSC-derived unless their lineage origin has been directly demonstrated.

High-dimensional tissue imaging provides tissue- and protein-level validation of these spatial-omics findings. Technologies such as multiplex immunofluorescence and imaging mass spectrometry can resolve multicellular neighborhoods comprising CAFs, T cells, myeloid cells, and vasculature while preserving tissue architecture (132). Studies in patients with HCC have further identified distinct spatial immune architectures and shown their associations with outcomes following ICI therapy. In resectable HCC and in patients treated with atezolizumab plus bevacizumab, the spatial organization of immune components, including T and B cells, has also been associated with clinical outcomes and therapeutic responses (133, 134). Thus, compared with simply quantifying immune cell abundance, cellular proximity and local ecological niches may provide greater clinical interpretive value. However, these tissue-level associations remain insufficient to define the lineage-specific contribution of HSC-derived CAFs without complementary lineage-resolved or functional validation.

Digital pathology and artificial intelligence offer further opportunities to translate these complex spatial features into clinically applicable stratification markers. Computational pathology studies based on HCC tissue sections have been able to quantify immune distributions across tumor–stroma and center–margin regions and identify spatial immune phenotypes such as inflamed, immune-excluded, and immune-desert patterns (135). In patients receiving atezolizumab plus bevacizumab, machine learning-based assessment of immune infiltration from pathological images has also shown potential for predicting therapeutic outcomes (136). However, spatial omics, high-dimensional imaging, and digital pathology remain limited by sample size, spatial resolution, regional sampling, platform heterogeneity, lack of analytical standardization, and insufficient multicenter external validation. Their clinical predictive value therefore requires confirmation in prospective studies.

In the context of ICI therapy, increasing attention has been paid to the importance of the spatial immune state in HCC. Multi-omics studies have shown that the distribution of CD8^+^ TRM cells across the tumor core, invasive margin, and adjacent tissues, as well as their dynamic changes after treatment, are associated with immunotherapy response (137). This suggests that ICI efficacy depends not only on the presence of T cells but also on their functional state and spatial localization. In HCC with highly activated CAF-rich stroma, ECM barriers and associated immunoregulatory networks may further restrict effector T-cell entry and functional recovery. However, overall CAF enrichment cannot be simply equated with ICI resistance, and its predictive value needs to be interpreted in the context of specific CAF states, spatial organization, and the broader multicellular ecosystem. Moreover, current clinical evidence does not support interpreting CAF enrichment itself as a surrogate marker for HSC-derived CAF abundance.

Accordingly, future patient stratification in HCC may need to move beyond single tumor or immune markers toward integrated assessment of stromal, immune, and spatial features. CAF molecular and transcriptional states, CD8^+^ T-cell infiltration and exhaustion or tissue-resident characteristics, as well as CAF–T-cell spatial distance, tumor–stroma boundaries, and immune-exclusion patterns, may all have potential value. Recent single-cell and spatial studies have begun to characterize the HCC immune microenvironment across multiple dimensions and explore its relationships with prognosis and therapeutic response (138). However, these models are still derived mainly from retrospective or single-center studies and require standardized, multicenter, and prospective validation before they can be incorporated into clinical decision-making. Where possible, future stratification models should also integrate fibroblast lineage information to distinguish HSC-derived CAFs from other CAF populations rather than relying solely on bulk CAF abundance or conventional stromal markers.

CAF-targeted therapy faces similar translational challenges. At present, there is no established HSC-derived CAF-specific therapy in HCC, with major limitations including heterogeneity in CAF origin and function, insufficient target specificity, and a restricted safety window in the setting of fibrosis or cirrhosis. Importantly, not all CAFs are uniformly tumor promoting. A recent study showed that FMO2^+^ CAFs can promote CD8^+^ T-cell infiltration and tertiary lymphoid structure formation through a CCL19–CCR7-related mechanism and are associated with improved responses to anti-PD-1 therapy (139). The lineage origin of such transcriptionally defined CAF states should, however, be distinguished from their functional phenotype and should not be assumed to be HSC-derived without direct evidence. Therefore, a more rational future strategy may not be broad CAF depletion, but rather the identification of CAF subsets that truly drive T-cell exclusion and therapeutic resistance according to lineage origin, functional state, and spatial localization, followed by selective inhibition, functional reprogramming, or stromal normalization in precise combination with ICIs and other microenvironment-targeted strategies.

## 6 Therapeutic strategies targeting HSC-derived CAF–T cell crosstalk

**Figure 3:**
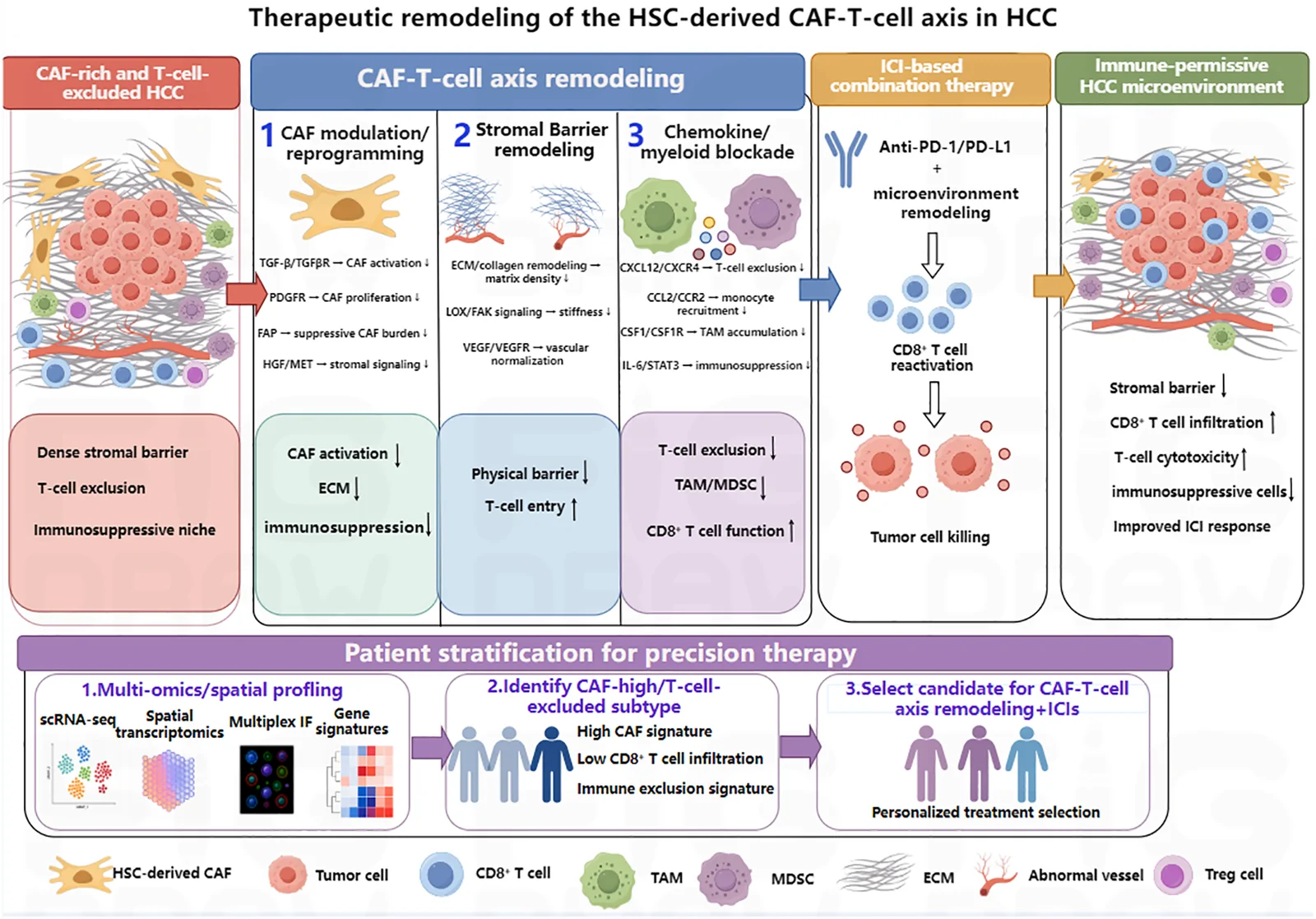
Therapeutic remodeling of the HSC-derived CAF–T-cell axis in HCC. This image summarizes a conceptual therapeutic framework for remodeling the CAF–T-cell axis in CAF-rich and T-cell-excluded HCC. Such tumors may exhibit dense stromal barriers, limited CD8^+^ T-cell infiltration, accumulation of immunosuppressive cells, and reduced responsiveness to immune checkpoint blockade. Potential therapeutic approaches include three complementary levels of microenvironmental remodeling. First, CAF modulation or functional reprogramming may involve TGF-β/TGFBR, PDGFR, FAP, HGF/MET, and related stromal pathways, with the aim of reducing persistent CAF activation, excessive ECM production, and immunosuppressive signaling rather than indiscriminately depleting all CAFs. Second, stromal-barrier remodeling may involve controlled ECM/collagen remodeling, modulation of abnormal mechanotransduction and matrix stiffness, and VEGF/VEGFR-related vascular normalization to improve T-cell entry into the tumor. Third, targeting chemokine and myeloid regulatory networks, including CXCL12/CXCR4, CCL2/CCR2, CSF1/CSF1R, and IL-6/STAT3, may reduce T-cell exclusion and TAM/MDSC-associated immunosuppression and thereby improve CD8^+^ T-cell function. These approaches may be mechanistically complementary to anti-PD-1/PD-L1 therapy by facilitating T-cell reinvigoration and tumor-cell killing. Patient selection may further integrate single-cell and spatial profiling, multiplex imaging, CAF/stromal signatures, CD8^+^ T-cell infiltration, and immune-exclusion features to identify CAF-high/T-cell-excluded HCC that may be more suitable for CAF–T-cell axis remodeling combined with ICIs. Unless otherwise specified, the cytokines, chemokines, and other mediators depicted in this image should not be interpreted as being exclusively derived from HSC-derived CAFs. Several signals, including IL-6, CCL2, and VEGF, may originate from immune cells, endothelial cells, hepatocytes, and other stromal populations within the HCC microenvironment. Several of the therapeutic strategies illustrated remain preclinical or translational rather than established clinical therapies in HCC.

HSC-derived CAFs may contribute to immune evasion in HCC through stromal barriers, immunosuppressive signaling, and spatial remodeling; however, many of the relevant pathways are shared across multiple CAF, stromal, immune, endothelial, and tumor-cell populations. Accordingly, therapeutic interventions should not focus solely on enhancing T-cell activation or on indiscriminate depletion of HSC-derived CAFs, but should instead address the coordinated regulation of CAF functional states, the ECM, abnormal vasculature, and compensatory immunosuppressive networks. **Figure 3** summarizes the major therapeutic strategies aimed at remodeling the broader CAF–T-cell axis, including selective CAF modulation and functional reprogramming, stromal normalization, blockade of chemokine and myeloid immune pathways, and combination therapy with ICIs. To further integrate these mechanisms with their potential translational value, **Table 2** summarizes representative CAF-associated mediators and stromal regulatory mechanisms, major molecular targets, their effects on T-cell biology, corresponding therapeutic strategies, current evidence, and major limitations.

**Table 2:** Representative CAF-associated mediators and stromal mechanisms linking T-cell regulation to potential therapeutic strategies in HCC.

CAF-associated mediator/mechanism	Representative CAF state	Major molecular target/signaling axis	Effects on T-cell biology	Corresponding therapeutic strategy	Current evidence	Key limitations	References
TGF-β1	Activated HSC-derived CAFs and myCAF-like states; specific subsets remain incompletely defined	TGFBR2/TGFBR1–SMAD2/3–SMAD4	Suppresses CD8^+^ T-cell effector function, promotes Treg-associated immune tolerance, and may further aggravate spatial exclusion by enhancing matrix production	Modulation of the TGF-β/TGFBR pathway; combination with PD-1/PD-L1 blockade	The mechanistic basis for TGF-β/SMAD-mediated HSC activation and fibrosis is relatively strong, while HCC studies support its involvement in stromal–immune regulation. Current translational studies increasingly emphasize combined targeting of the TGF-β-related stromal–immune network and ICIs.	TGF-β has pleiotropic effects and multiple cellular sources; systemic blockade may impair liver homeostasis, regeneration, and tissue repair. Direct HSC-derived CAF-specific causal evidence in human HCC remains limited, and HSC-derived CAF-specific targeting is not currently feasible.	(52–55, 140, 141)
CXCL12	Peritumoral apCAF-like or inflammation-associated CAF states; lineage origin remains incompletely defined	CXCL12–CXCR4 and downstream migratory signaling	Alters CD8^+^ T-cell chemotaxis and spatial localization, promotes T-cell marginalization, and restricts infiltration into the tumor parenchyma	CXCL12/CXCR4 blockade; combination with ICIs or stromal modulation	HCC spatial transcriptomic and tissue studies support an association between CAF spatial states, CXCL12/CXCR4-related signaling, and T-cell exclusion.	CXCL12 is produced by multiple stromal and immune populations; CAF lineage specificity remains unresolved, and direct therapeutic causality in HCC remains limited. Systemic targeting may also disrupt physiological lymphocyte trafficking.	(23, 56–58)
IL-6	iCAF-like inflammatory states; CAF-source specificity has not been fully established	IL-6R/gp130–JAK1/2–STAT3–c-MAF	Promotes CD8^+^ T-cell dysfunction and exhaustion-associated states and reinforces local inflammatory immunosuppression	IL-6/IL-6R blockade; JAK/STAT3 inhibition; combination with ICIs	HCC patient samples and functional studies support the involvement of IL-6/STAT3 signaling in CD8^+^ T-cell dysfunction and inflammatory immunosuppression.	IL-6 originates from multiple cellular sources and cannot be specifically attributed to HSC-derived CAFs. Current strategies do not enable CAF-specific IL-6 blockade and may produce broad immunological effects.	(59–62)
CCL2 and related myeloid regulation	Activated HSC/CAF-like cells and other microenvironmental cells	CCL2–CCR2; HSC–CX3CR1^+^ macrophage-associated regulation	Recruits and reprograms immunosuppressive myeloid cells, thereby indirectly impairing CD8^+^ T-cell proliferation and antitumor activity	CCL2/CCR2 and myeloid-cell modulation; combination with ICIs	Substantial evidence supports CCL2/CCR2-mediated myeloid immune regulation, and recent HCC studies provide functional evidence for HSC–macrophage–CD8^+^ T-cell interactions.	CCL2 arises from multiple cellular sources, and pathway redundancy or compensatory myeloid-recruitment mechanisms may limit the durability and single-agent efficacy of CCL2/CCR2 blockade.	(63–66)
Collagen, fibronectin, and other ECM components	FAP^+^, matrix-producing CAFs and myCAF-like states	Integrin/FAK-related mechanotransduction, matrix stiffness, and other ECM-sensing signals	ECM densification and increased tissue stiffness create structural and mechanical barriers that restrict CD8^+^ T-cell migration and infiltration into the tumor parenchyma	Matrix normalization; modulation of abnormal mechanotransduction and matrix-production programs; combination with ICIs	HCC single-cell, spatial-omics, tissue, and preclinical studies support associations of FAP^+^ and matrix-producing CAFs with ECM remodeling and spatial immune exclusion.	Evidence for matrix normalization remains predominantly preclinical and mechanistic. FAP and related stromal markers are not HSC-lineage specific, and excessive ECM reduction may compromise tissue integrity, particularly in cirrhosis.	(21, 24, 67–69, 142–146)
VEGFA and CAF-associated angiogenic programs	VEGFA^+^ CAFs; lineage origin remains incompletely defined	Hypoxia/HIF-1α–VEGFA–VEGFR2	Indirectly restricts effector T-cell recruitment and infiltration through abnormal vasculature, poor perfusion, and hypoxia, while reinforcing an immunosuppressive microenvironment	Anti-VEGF/VEGFR therapy and vascular normalization; combination with ICIs and stromal modulation	HCC spatial-omics studies show that VEGFA^+^ CAFs are preferentially enriched within tumor regions and are associated with hypoxic and angiogenic programs.	VEGFA is produced by multiple cell types and is not CAF specific. Antiangiogenic therapy should not be equated with direct CAF targeting and may also affect normal hepatic vascular homeostasis.	(22, 70–72)
CCL19	FMO2^+^ immune-supportive CAFs	CCL19–CCR7	Promotes CD8^+^ T-cell infiltration, tertiary lymphoid structure formation, and local immune activation and is associated with improved response to anti-PD-1 therapy	Preservation or enhancement of the immune-supportive program of FMO2^+^ CAFs; exploration of CCL19-related immune enhancement in combination with ICIs	Evidence from HCC patients, multi-omics analyses, coculture experiments, and *in situ* models supports an immune-supportive FMO2^+^ CAF state associated with enhanced T-cell infiltration and improved anti-PD-1 response.	The lineage origin of FMO2^+^ CAFs remains incompletely defined, and therapeutic preservation or enhancement of this program remains exploratory. This axis also highlights the functional duality of CAFs and argues against indiscriminate CAF depletion.	(25, 139)

### 6.1 Selective CAF targeting and functional reprogramming

HSC-derived CAFs represent an important component of the immunosuppressive stromal microenvironment in HCC, particularly in the context of chronic liver injury and fibrosis, but they do not constitute the entire CAF population. Persistent activation of profibrotic and immunosuppressive CAF states can promote ECM deposition, T-cell exclusion, and local immunosuppression (118, 119, 123). Accordingly, TGF-β/TGFBR, PDGFR, FAP, HGF/MET, and other stromal or mechanotransduction-related pathways have all been considered potential therapeutic targets (147–149). These pathways, however, are not specific to HSC-derived CAFs and may also operate in other fibroblast subsets or non-fibroblast components of the HCC microenvironment. The rationale underlying these strategies is therefore not simply to reduce CAF abundance, but rather to weaken pathological stromal programs that persistently maintain profibrotic, immunosuppressive, and T-cell-excluding states.

CAFs do not constitute a functionally uniform protumor cell population. Different CAF subsets may contribute to matrix production, inflammatory regulation, immunosuppression, and tissue repair, and some states may even exert tissue-restraining or potentially immune-supportive functions (150). Therefore, indiscriminate CAF depletion may not only fail to produce durable therapeutic benefit but may also disrupt liver homeostasis, tissue repair, and regeneration mediated by normal HSCs and other fibroblast populations. At the same time, commonly used markers such as FAP, α-SMA, and PDGFR cannot reliably distinguish HSC-derived CAFs from other CAF subsets or normally activated HSCs, making lineage and cell-state specificity major obstacles to direct CAF targeting. CAFs also exhibit substantial state plasticity, and inhibition of a particular subset or signaling pathway may be followed by phenotypic switching or activation of alternative signaling programs that re-establish an immunosuppressive stromal ecosystem.

The therapeutic concept of CAF targeting is therefore gradually shifting from nonselective depletion toward functional reprogramming and stromal normalization. The goal is to identify CAF states that genuinely contribute to T-cell exclusion and therapeutic resistance and to suppress persistent profibrotic or immunosuppressive programs while preserving normal stromal homeostasis and tissue-repair functions in the liver as much as possible. In principle, this approach may be better suited than broad CAF depletion to HCC, which commonly develops in the setting of fibrosis or cirrhosis. However, specific targets and clinically validated biomarkers capable of reliably distinguishing protumor HSC-derived CAFs from other CAF populations, protective stromal cells, or normally activated HSCs are still lacking.

### 6.2 Stromal normalization and restoration of T-cell infiltration

CAF-associated ECM remodeling and an abnormal mechanical microenvironment are important factors restricting the entry of effector T cells into the HCC tumor parenchyma. Strategies aimed at restoring T-cell infiltration may therefore target pathological stromal architecture and its associated regulatory networks. Current potential approaches include reducing abnormal collagen deposition and matrix crosslinking, modulating mechanotransduction, and targeting microenvironmental signals associated with chemotaxis, myeloid immunosuppression, and abnormal vasculature (142, 143). Compared with complete elimination of the tumor stroma, a more rational therapeutic goal may be stromal normalization, namely, the controlled reduction of pathological ECM accumulation and tissue stiffness to improve T-cell migration, vascular function, and drug penetration while preserving as much as possible the matrix required to maintain hepatic structural integrity.

Recent studies have provided further support for stromal normalization strategies. Notch-related signaling can contribute to HSC/myofibroblast activation and has also been implicated in CAF-mediated ECM remodeling in preclinical models. In particular, the Notch3–Hes1 axis is associated with the expression of matrix-related molecules such as α-SMA and COL1A1, suggesting that aberrant Notch signaling may represent an important regulatory node linking HSC activation, ECM deposition, and stromal remodeling (144). Meanwhile, recent studies of CAF-mediated immune evasion have further shown that CAF-associated ECM barriers and signaling networks involving TGF-β, CXCL12, inflammatory mediators, and myeloid immunosuppression can cooperate to establish an immune-excluded ecosystem, restricting CD8^+^ T-cell entry into the tumor parenchyma and reducing the efficacy of ICIs (145, 146). Because these mediators are not uniquely derived from HSC-derived CAFs, they should be interpreted as components of a broader multicellular stromal–immune network.

It should be noted that evidence for stromal normalization strategies in HCC remains largely preclinical, and the optimal intensity of intervention and therapeutic safety window have yet to be established. Because HCC commonly arises in the setting of liver fibrosis or cirrhosis, excessive disruption of the ECM may compromise the structural stability of liver tissue and potentially increase risks in patients with portal hypertension or impaired liver function. Future studies therefore need to define therapeutically relevant CAF and ECM states, the appropriate degree of stromal remodeling, and corresponding patient-selection biomarkers to achieve a balance between relieving immune exclusion and preserving hepatic tissue homeostasis. Lineage-resolved approaches will also be required to determine when HSC-derived CAFs themselves constitute a therapeutically actionable stromal population.

### 6.3 Combination immunotherapy, patient stratification, and translational challenges

From a clinical translational perspective, CAF- or stroma-related interventions are more likely to serve as combination modulatory strategies for immunotherapy than as standalone treatments. PD-1/PD-L1 blockade can relieve some forms of T-cell functional suppression, but CAF-associated stromal barriers, ECM accumulation, TGF-β/CXCL12-related signaling, abnormal vasculature, and immunosuppressive myeloid cells in HCC can still collectively restrict effector T-cell infiltration and functional recovery. Recent studies have further suggested that resistance to immunotherapy in HCC is commonly maintained by multiple layers of mechanisms, including stromal barriers, myeloid immunosuppression, metabolic reprogramming, and alternative immune checkpoints. Accordingly, CAF functional reprogramming, modulation of the stromal and vascular microenvironment, and myeloid immune interventions may provide mechanistic complementarity to PD-1/PD-L1 blockade (140).

This concept has also driven HCC immunotherapy from single-pathway blockade toward multidimensional combination strategies. Recent studies have shown that combining antiangiogenic agents or multitargeted tyrosine kinase inhibitors with ICIs can enhance therapeutic efficacy by simultaneously modulating tumor vasculature and the immune microenvironment, while combination strategies targeting CAFs, TAMs, TANs, and other immunosuppressive components are also emerging as approaches for overcoming ICI resistance (141). Meanwhile, preclinical studies in HCC have demonstrated that remodeling the tumor immune microenvironment and enhancing CD8^+^ T-cell infiltration and function can further increase sensitivity to anti-PD-1 therapy, providing experimental support for combining microenvironmental modulation with ICIs (151). However, these findings should be interpreted as evidence for broader microenvironmental modulation rather than as validation of HSC-derived CAF-specific targeting.

Mechanistic rationale does not necessarily translate into clinical benefit. The TGFBR1 inhibitor galunisertib in combination with the anti-VEGFR2 antibody ramucirumab was evaluated in a Phase 1b study in advanced HCC. Although the combination was generally tolerable, its antitumor activity was limited and did not support further clinical development (152). This finding indicates that simultaneous blockade of stromal- and vascular-related signaling is not sufficient to guarantee therapeutic success and that patient selection, the degree of tumor dependence on the targeted pathways, and alternative immunosuppressive mechanisms may all influence treatment efficacy.

Meanwhile, strategies that more directly combine stromal–immune regulation with checkpoint blockade are entering clinical development in HCC. Livmoniplimab is a monoclonal antibody that targets the GARP: TGF-β1 complex and restricts the release of active TGF-β1. Its combination with the anti-PD-1 antibody budigalimab has demonstrated manageable safety and preliminary antitumor activity in a Phase 1 study of advanced solid tumors (153). In HCC, LIVIGNO-2 (NCT06109272) is an ongoing randomized Phase 2/3 study evaluating livmoniplimab plus budigalimab in patients with locally advanced or metastatic HCC who have not received prior systemic therapy. Another Phase 1b study in China (NCT06487559) is evaluating the same combination in patients with Child-Pugh A locally advanced or metastatic HCC who experienced disease progression after first-line ICI-containing therapy. Importantly, these treatments are not specific interventions targeting HSC-derived CAFs. Rather, they indirectly influence the CAF–T-cell ecosystem by modulating the TGF-β-related immune–stromal network. They should therefore be regarded as clinical tests of combined stromal–immune modulation rather than as evidence that HSC-derived CAF-specific therapy has entered a mature stage of clinical development.

Not all patients with HCC are likely to benefit from combined microenvironmental interventions. Responses to immunotherapy in HCC exhibit substantial molecular and immune heterogeneity, with marked interpatient differences in PD-L1 regulation, T-cell states, stromal organization, and local immune composition (154). More rational patient selection may therefore require integrated assessment of CAF/stromal activation, CD8^+^ T-cell infiltration and exhaustion states, myeloid immunosuppression, and alternative immune checkpoint features rather than reliance on a single biomarker. Recent studies of patients with HCC receiving anti-PD-1 therapy combined with targeted agents have further shown that multiparametric immune phenotyping can provide additional prognostic and therapeutic stratification information, supporting the development of composite biomarker systems based on tumor microenvironmental states (155).

Future clinical studies also need to establish an actionable stromal–immune stratification system. Potential markers may include CAF-associated features such as FAP, α-SMA, COL1A1, and PDGFRβ; CD8^+^ T-cell infiltration; T-cell state markers such as PD-1, TIM-3, and TOX; and tissue architectural features including the spatial distance between CAFs and CD8^+^ T cells, fibrous septa, and immune-exclusion patterns. Because these conventional CAF markers are not lineage specific, their use should ideally be complemented by lineage-resolved molecular or spatial information when the therapeutic hypothesis specifically concerns HSC-derived CAFs. Integration of multiplex immunofluorescence, spatial transcriptomics, single-cell sequencing, lineage-informed profiling, and digital pathology may help identify patients whose immune evasion is truly dependent on particular CAF–T-cell ecosystems and thereby guide precise combinations of CAF/stromal modulation with ICIs.

Beyond patient selection, the underlying liver disease characteristic of HCC remains an important barrier to clinical translation. Most patients have varying degrees of liver fibrosis or cirrhosis, and some also present with portal hypertension, abnormal sinusoidal architecture, heterogeneous tumor perfusion, and reduced hepatic functional reserve. These factors may not only limit drug delivery and penetration into stromal regions of the tumor but may also narrow the safety window for CAF- or ECM-targeted interventions. At the same time, CAF-related networks exhibit substantial redundancy and plasticity. Inhibition of a single pathway may be compensated for through alternative chemotactic signals, inflammatory pathways, myeloid cell recruitment, or stromal remodeling, while therapeutic pressure may also induce transitions in CAF states and re-establish an immunosuppressive ecosystem. Therefore, future CAF-related therapies should shift from single-target blockade toward precision reprogramming based on CAF lineage, functional state, spatial localization, and dynamic biomarkers, combined with mechanistically complementary therapies to reduce compensatory immunosuppression and acquired resistance.

## 7 Conclusions and future perspectives

Bidirectional crosstalk between HSC-derived CAFs and T cells provides an organ-specific immune–stromal framework linking chronic liver injury, fibrotic stromal remodeling, HCC immune evasion, and therapeutic resistance. Current evidence indicates that CAF-associated stromal remodeling, immunoregulatory signaling, and spatial organization are closely associated with restricted T-cell infiltration, functional impairment, and poor therapeutic responses, while distinct T-cell subsets can in turn reshape HSC and CAF states. However, these observations remain insufficient to establish a complete lineage-resolved causal model. A central challenge is therefore to determine which HSC-derived CAF states are truly required for T-cell exclusion, at which disease stages and within which spatial niches these effects occur, and which stromal–immune interactions are necessary to sustain immune evasion and therapeutic resistance.

First, the lineage origin, functional state, and spatial localization of CAFs remain fundamental unresolved issues. Although single-cell and spatial-omics studies have identified multiple CAF states, transcriptionally defined CAF subsets cannot be directly equated with HSC-derived CAFs, and the HSC-lineage populations that consistently drive T-cell exclusion have yet to be clearly defined. CAF function is also highly context dependent and plastic. Some CAF states promote matrix accumulation and immunosuppression, whereas others may contribute to tissue restraint, repair, or even local immune support. Future studies should therefore distinguish cellular origin from functional state and determine whether these phenotypes undergo reversible transitions during chronic liver injury, tumor initiation, progression, and therapeutic pressure. The reverse influence of T cells on stromal populations is similarly unlikely to be uniform. Different CD4^+^ and CD8^+^ T-cell states, as well as γδ T cells, MAIT cells, and TRM cells, may exert distinct or even opposing effects on HSCs and CAF populations at different disease stages, yet direct causal evidence for many of these interactions in human HCC remains limited.

Addressing these questions will require a transition from descriptive omics toward spatiotemporal and lineage-resolved causal validation. Future studies should integrate lineage tracing, conditional gene editing, and cell subset-specific perturbation with single-cell sequencing, spatial transcriptomics, multiplex immune imaging, and spatial metabolic analyses to resolve cellular origin, spatial organization, and functional dependence simultaneously. Longitudinal animal models will be particularly important for tracking the transition of HSCs into distinct CAF-like states together with changes in T-cell differentiation and exhaustion over the course of disease. In parallel, patient-derived organoids, tumor slice cultures, and CAF–T-cell coculture systems can provide human experimental platforms for validating candidate communication pathways. Signals identified by ligand–receptor inference or spatial colocalization should be subjected to functional testing through blockade, knockout, rescue, or spatial perturbation rather than being assigned causal significance solely on the basis of computational association. Paired pre- and post-treatment samples and longitudinal sampling will also be essential for determining whether immune checkpoint blockade or other therapies induce CAF state transitions, compensatory signaling, or the emergence of new immunosuppressive niches.

Future therapeutic development should also extend beyond direct CAF targeting toward simultaneous restoration of T-cell competence and disruption of multicellular immunosuppressive networks. One emerging approach is mRNA-based immune rejuvenation. Transient hepatic delivery of mRNAs encoding thymic trophic factors such as DLL1, FLT3L, and IL-7 has recently shown the capacity to enhance lymphoid progenitor expansion and thymopoiesis, replenish peripheral naïve T-cell pools, restore antigen-specific CD8^+^ T-cell responses, and improve responsiveness to immune checkpoint blockade in aged preclinical models (156). Although this strategy has not yet been specifically validated in HCC, restoration of T-cell fitness may provide a complementary therapeutic dimension to CAF functional reprogramming or stromal normalization, particularly when persistent T-cell dysfunction limits the effectiveness of microenvironment-targeted interventions.

At the same time, the CAF–T-cell axis should increasingly be considered within a broader multicellular network that includes tumor-associated macrophages (TAMs). TAMs are closely involved in CD8^+^ T-cell dysfunction and exhaustion in HCC and may act as an important intermediary between stromal remodeling and adaptive immune suppression (157). Of particular interest, SPP1^+^ macrophages have been shown to spatially interact with CAFs at the tumor boundary, promote ECM remodeling, and contribute to the formation of a tumor immune barrier that restricts cytotoxic T-cell infiltration into the tumor core. Experimental disruption of SPP1-related macrophage–CAF interactions can reduce CAF accumulation and improve the efficacy of PD-1 blockade in preclinical HCC models (158). These observations support a shift from a binary CAF–T-cell model toward a coordinated CAF–TAM–T-cell framework in which stromal remodeling, myeloid immunosuppression, and T-cell dysfunction mutually reinforce one another. Future therapeutic strategies may therefore benefit from simultaneous or sequential modulation of pathogenic CAF states, immunosuppressive TAM programs, and dysfunctional T-cell states rather than targeting any single compartment in isolation. Importantly, the CAF populations involved in such multicellular niches should not automatically be assumed to be HSC-derived unless supported by lineage-resolved evidence.

The key challenge for clinical translation is to move from broad CAF targeting toward stratifiable, monitorable, and reversible stromal–immune regulation. Future clinical studies should prioritize composite biomarker systems capable of identifying CAF-enriched/T-cell-excluded HCC by integrating CAF subset characteristics, ECM states, CD8^+^ T-cell infiltration and exhaustion, myeloid-cell composition, spatial relationships among CAFs, TAMs, and T cells, digital pathology, spatial omics, and circulating biomarkers. Where therapeutic hypotheses specifically concern HSC-derived CAFs, lineage-resolved information should be incorporated rather than relying solely on conventional CAF markers or bulk stromal enrichment scores. Therapeutic development should also define both the appropriate patient population and the optimal intervention window. Compared with nonselective CAF depletion, greater priority should be given to functional reprogramming, stromal normalization, restoration of T-cell competence, and mechanistically complementary combinations with ICIs or myeloid-directed therapies. At the same time, liver fibrosis, cirrhosis, portal hypertension, and reduced hepatic functional reserve must be incorporated into drug-delivery strategies, dose optimization, and safety assessments to avoid disrupting normal hepatic homeostasis while attempting to relieve tumor stromal constraints.

Overall, HSC-derived CAF–T-cell crosstalk should be viewed as one component of a broader, spatially organized, and dynamically evolving immune–stromal network in HCC. The next stage of research should move from cataloguing additional associated molecules toward defining critical cellular states and their causal dependencies; from static tissue descriptions toward longitudinal, lineage-resolved, and spatially resolved analyses; and from nonselective stromal intervention toward precision remodeling of coordinated CAF, myeloid, and T-cell programs. Integrating lineage-specific mechanistic validation, human experimental systems, longitudinal spatial analyses, immune-rejuvenation strategies, and prospective clinical studies will be essential for determining the specific contribution of HSC-derived CAFs within the broader CAF ecosystem and for establishing whether these multicellular stromal–immune networks can ultimately be translated into actionable biomarkers and therapeutic targets for patients with HCC.
